# Chiropractic students’ views on needle stick injuries and protocols at a South African university

**DOI:** 10.4102/hsag.v30i0.2868

**Published:** 2025-04-30

**Authors:** Fatima Ismail, Connor Mcleod

**Affiliations:** 1 Department of Chiropractic, Faculty of Health Sciences, University of Johannesburg, Johannesburg, South Africa

**Keywords:** Chiropractic, student, needle stick injury, education, knowledge, perception

## Abstract

**Background:**

Needle stick injuries (NSIs) pose serious occupational risks for health professionals and students, with psychological and physical consequences. Despite increased awareness, NSIs persist. Research focussing on chiropractic students’ perspectives on NSIs and related protocols is limited, in South Africa and internationally.

**Aim:**

This study aimed to evaluate the knowledge, attitudes and perceptions of chiropractic students towards NSIs and related protocols.

**Setting:**

This study was undertaken at the Chiropractic Department, University of Johannesburg.

**Methods:**

A descriptive, cross-sectional, quantitative study involving an adapted anonymous online questionnaire was distributed to undergraduate Bachelor of Health Sciences and postgraduate Master of Health Sciences (BHSc and MHSc) chiropractic students. Data were analysed using frequencies, descriptive statistics and cross-tabulations to identify relationships in the data.

**Results:**

The respondents (*n* = 107; 42% response rate) had a mean age of 22.83 years and were mostly females (76.6%), which showed high NSI knowledge (88.58%; s.d. = 9.455); however, postgraduates scored higher overall (*p* < 0.001). While both groups were well informed about risks and protocols, undergraduates emphasised the need for Hepatitis B vaccination (*p* = 0.021) and reporting of unused sterile needle injuries (*p* = 0.010), further highlighting variances between the cohorts.

**Conclusion:**

Chiropractic students exhibited good NSI knowledge, postgraduates more so, but attitudes and reporting behaviours’ varied. Enhanced, standardised education on the urgency of reporting NSIs is recommended to improve protocol and safety practices. Future research should explore long-term NSI protocol adherence.

**Contribution:**

This study provides important baseline South African data on perspectives of NSI in a chiropractic student cohort.

## Introduction

Research on needle stick injuries (NSIs) has predominantly focussed on their prevalence, associated infections and the effectiveness of post-exposure management protocols (Al-Mugheed et al. 2023; Thekkiniyakath Ali et al. 2023). Despite widespread awareness, the global prevalence of NSIs remains alarmingly high, with over two million injuries reported annually (Bouya et al. 2020). This risk is particularly elevated among students in the health professions, who, because of their limited experience and knowledge, are more susceptible to these injuries (Datar et al. 2022). Estimates indicate that up to 47% of students may experience an NSI during training (Dehvan et al. 2021). Most NSIs can be avoided through proper instrument handling, adherence to safety precautions and a solid understanding of post-exposure prophylaxis (PEP) (Datar et al. 2022).

Studies have identified a gap between healthcare personnel’s knowledge and the actual practice of NSI protocols (Al-Mugheed et al. 2023), suggesting potential deficiencies in educational programmes and institutional enforcement (Keicher et al. 2024). While some healthcare institutions report adequate knowledge and positive attitudes towards NSI protocols, they also highlight a shortfall in the practical application of these protocols, particularly among medical students and healthcare workers (Alsabaani et al. 2022). When considering these types of knowledge, attitude and perception studies, and in the context of this current study, knowledge relates to what students factually know about the topic, while attitudes and perceptions relate to what the students believe about the topic (Andrade et al. 2020). Research has shown that healthcare students often lack sufficient knowledge of infection prevention practices, especially in NSI prevention and hand hygiene (Kulkarni et al. 2013).

The high prevalence of NSIs among student cohorts is further complicated by the underreporting of such occupational injuries, driven by factors such as the perception that the injury is not severe enough to report, social stigma and the burden of reporting procedures (Zhang et al. 2018). This underreporting can have serious consequences, as it often results in students not receiving proper medical management. The lack of timely reporting also makes it difficult to monitor the true scale of NSIs and hinders the allocation of resources for prevention and treatment effectively (Tonghui et al. 2023). Studies show that a correlation between socio-economic development and NSI reporting rates exists; while low-income countries have a 75.0% underreporting rate, middle-to-high-income countries, such as South Africa, and high-income countries have underreporting rate of 61.5% and 52.4%, respectively (Behzadmehr et al. 2023).

These injuries pose serious risks, including psychological distress (Hambridge, Nichols & Endacott 2016) and blood-borne infections, especially in countries such as South Africa, where infectious diseases are exceedingly prevalent (Nxasana et al. 2022). Hepatitis B Virus (HBV), Hepatitis C Virus (HCV) and human immunodeficiency virus (HIV) carry varying transmission risks associated with NSIs where HBV has the highest risk (6% – 30%), followed by HCV (1.8%) and HIV (0% – 10%) (Hussain et al. 2012). A review of the literature showed that medical students who experience sharps injuries often suffer from a range of emotional and psychological effects, including anxiety, stress, depression and even post-traumatic stress disorder. These injuries can also evoke feelings of embarrassment, fear, anger and a significant loss of confidence necessitating psychological support and counselling to assist with coping with the emotional toll of such incidents (Hambridge 2022; Hambridge et al. 2016).

Despite the considerable risks, students continue to demonstrate inadequate compliance with standard precautions and possess limited knowledge of sharp instruments and NSI prevention and management (Al Qadire et al. 2021). These risks associated with NSIs highlight the critical need for enhanced education and self-directed learning programmes to improve students’ understanding and awareness of NSI prevention and management and reducing NSI occurrence (Cheetham et al. 2021). There is an abundance of research available on the perspectives of NSIs held by health students such as medical, dental and nursing students (Datar et al. 2022; Hambridge et al. 2016; Musekene, Modjadji & Madiba 2020); however, there is a significant gap in understanding NSI perspectives within the chiropractic profession and its students. The chiropractic qualification comprises a non-exit level 4-year BHSc followed by a 2-year MHSc degree. The chiropractic students are exposed to needles or sharp instruments throughout their academic years, beginning with cadaver dissection in the 2nd year of the BHSc programme, advancing to myofascial dry needling in the 4th year of BHSc and 1st year of MHSc and extending to clinical exposure to patients and hands-on training during the 1st and 2nd years of their MHSc qualifications.

Needle stick injury training is offered comprehensively in the 4th year of the BHSc programme within the Myofascial Dry Needling module (UJ 2024). Given that myofascial dry needling is a component of the chiropractic curriculum and scope of practice (ed. Ahpcsa 2020), it is essential to address this gap. This study’s objectives addressed this gap by assessing the current level of knowledge regarding NSIs and associated protocols and attitudes towards NSI prevention and management practices of chiropractic students at a South African institution to understand their attitudes and perceptions of the importance of adhering to NSI-related protocols in clinical practice. This research is vital for improving safety protocols and reducing risks for chiropractic students and their patients.

## Research methods and design

### Study design, setting, population and sample size

A descriptive, cross-sectional quantitative study was conducted using a non-probability, voluntary and convenience response sampling method (Vehovar, Toepoel & Steinmetz 2016). The questionnaire was distributed via Google Forms (Google, Mountain View, CA, US) to chiropractic students at the University of Johannesburg (UJ), including undergraduate (1st to 4th year BHSc) and postgraduate (1st to 2nd year MHSc) students. This study was conducted at a single institution to gain a focussed understanding of the students’ perspectives on NSI and its related NSI protocols as provided by their institution. Ethical approval was obtained from the University of Johannesburg’s Faculty of Health Sciences (REC-2472-2023). With 255 students enrolled, 107 responded, yielding a 42% response rate and a margin of error of ±7.14% at a 95% confidence level.

### Data collection tool

The questionnaire was developed by adapting 19 questions from nine existing NSI studies (Alsabaani et al. 2022; Altaf et al. 2022; Azman et al. 2020; Jaber 2011; Joukar et al. 2018; Mazhar et al. 2020; Patel et al. 2018; Pavithran et al. 2015; Suliman et al. 2016) with 7 additional questions created by the researchers. Permission requests to use and modify these questions were sent to the main authors of the nine studies. The final questionnaire had 26 questions with three sections: Section A gathered demographic data, Section B assessed NSI knowledge with 14 true or false questions and Section C explored attitudes and perceptions with 9 items on a 5-point Likert scale (1-strongly disagree to 5-strongly agree). Each participant could submit only one response. The questionnaire’s content validity was ensured through the review by five faculty staff with expertise in NSI protocols and a statistician, confirming that all necessary dimensions of the construct were addressed. Context validity was assessed by conducting a pilot study within the target population, which included five postgraduate students, ensuring that the questionnaire applied to the specific academic setting. Face validity was confirmed through the pilot participant feedback, which indicated that the questionnaire appeared to measure the intended construct appropriately, and no changes were necessary (Roebianto et al. 2023).

### Data collection and analysis

The study was presented to undergraduate BHSc and postgraduate MHSc chiropractic classes by one of the researchers, where students were informed of the study purpose and details including what would be expected of them and the duration of their participation, which was 10–15 min. Potential participants were given the opportunity to ask questions following this. The link to the Google Form, including an information letter, consent form and questionnaire, was shared through class WhatsApp (Facebook Inc, Menlo Park, California, US) groups by the class representatives. Respondents were able to review and modify their answers, such as using a ‘back’ button. Data collection occurred from 06 March to 04 April 2024. Only fully completed questionnaires were analysed, and just one response per student was allowed. The anonymous responses were entered manually into an Excel file (Microsoft Corporation, 2024). All data will be stored in a password-protected file for the duration of 5 years, as no identifying data were collected all data are anonymous.

Data analysis was performed using Statistical Package for the Social Sciences (SPSS) version 26.0 (Statistical Product and Service Solutions, IBM, New York, US), focussing on basic frequency and descriptive statistics to summarise participant knowledge, attitudes and perceptions. Likert-scale items underwent exploratory factor analysis (EFA) to identify factor structures, and Cronbach’s alpha was calculated for reliability. Descriptive statistics provided insights into key variable patterns, while normality and comparison testing, including independent-samples T-tests, explored differences between undergraduate and postgraduate groups. Associations within the data were examined using cross-tabulation and Pearson chi-square tests, with a significance threshold set at *p* < 0.05 (Pallant 2007).

The internal consistency of Section C was assessed using Cronbach’s alpha and inter-item correlations for two distinct factors: Reporting Compliance and Protocol Awareness (Factor A; 0.688; 0.352) and Perception of Risk and Response NSIs (Factor B; 0.659; 0.395). This suggests a moderate level of agreement between the items. Although the factors demonstrate reasonable reliability, there is potential for improvement in aligning the items more closely with the underlying construct (Knekta, Runyon & Eddy 2019).

### Ethical considerations

All ethical guidelines from the University of Johannesburg Faculty of Health Sciences Research Ethics and Higher Degrees Committees were followed, with the required permissions obtained. The ethics approval number is (REC-2472-2023). Student confidentiality and anonymity were maintained by not collecting any identifying information. Participants were informed that their digital footprint would not be stored or used in a way that could identify them. The online information and consent forms stated that participation was voluntary and noted that participants could withdraw at any point before submission. Consent was indicated by clicking ‘agree and continue’. Informed consent was received from all participants. There were no associated risks or direct benefits, and the data were stored securely in a password-protected document for 5 years.

## Results

A total of 107 chiropractic students responded to the online questionnaire, from the total sample population of 255, yielding a 42% response rate. The mean age was 22.83 (± 3.725) years, with most of the student respondents being female (76.60%; *n* = 82). The largest group of respondents belonged to the 2nd year MHSc year of study (29.90%; *n* = 32). Table 1 presents the demographic characteristics of the respondents.

**TABLE 1:** Demographic characteristics of the student respondents.

Demographic characteristic	%	*n*
**Age (years)**		
18–20	22.4	24
21–23	44.9	48
24–26	22.4	24
27–29	2.8	3
30+	7.5	8
**Sex**		
Female	76.6	82
Male	23.4	25
**Year of study**		
**Total undergraduate**	57.9	62
1st year BHSc	11.2	12
2nd year BHSc	8.4	9
3rd year BHSc	19.6	21
4th year BHSc	18.7	20
**Total postgraduate**	42.1	45
1st year MHSc	12.1	13
2nd year MHSc	29.9	32

BHSc, Bachelor of Health Sciences; MHSc, Master of Health Sciences.

### Chiropractic student respondents’ knowledge of needle stick injuries and related protocols

The results from the questionnaire showed that undergraduate and postgraduate chiropractic students had good knowledge of NSIs and related protocols. Most students correctly identified NSIs as accidental needle punctures (92.5%, *n* = 99), recognised their preventability (98.1%, *n* = 105) and unanimously agreed on the need for immediate disposal of needles in a sharps bin (100.0%, *n* = 107). Postgraduate students had greater awareness of certain practices, such as promoting bleeding after injury (86.7%, *n* = 39) and washing the area with soap and water (95.6%, *n* = 43). Both groups were highly aware of the need to report NSIs immediately (98.1%, *n* = 98) and seek primary healthcare (91.5%, *n* = 98).

All respondents correctly identified HBV, HCV and HIV as common risks from NSIs (100.0%, *n* = 107). However, 13.0% (*n* = 14) mistakenly believed that sterile needles could transmit infections. While most understood the importance of PEP (93.4%, *n* = 100), only 66.3% (*n* = 71) agreed that anti-retroviral drugs are mandatory after an NSI. Reporting NSIs, even if the student injured had negative lab results, was recognised by 85.0% (*n* = 91) of students, with postgraduates being more informed (95.6%, *n* = 43). Further details can be seen in Table 2.

**TABLE 2:** Chiropractic student respondents’ knowledge of needle stick injury and its related protocols.

Item	True or false	Total sample		Undergraduate		Postgraduate	
		%	*n*	%	*n*	%	*n*
A NSI is defined as wounds caused by needles that accidentally puncture the skin.	T	92.5	99	90.3	56	95.6	43
	F	7.4	8	9.7	6	4.4	2
Needle stick injuries are preventable.	T	98.1	105	98.4	61	97.8	44
	F	1.8	2	1.6	1	2.2	1
Safer devices, techniques and gloves are needed to avoid needle stick accidents.	T	92.5	99	93.5	58	91.2	41
	F	7.4	8	6.5	4	8.8	4
Needles should be placed into a medical sharps bin immediately after use.	T	100.0	107	100.0	62	100.0	45
	F	0.0	0	0.0	0	0.0	0
You should promote active bleeding by squeezing the site of injury.	T	55.1	59	32.3	20	86.7	39
	F	44.8	48	67.7	42	1.3	6
Washing the area with soap and water is recommended to decrease the risk of infection immediately after experiencing a NSI.	T	80.3	86	69.4	43	95.6	43
	F	19.6	21	30.6	19	4.4	2
A NSI should be reported immediately.	T	98.1	105	98.4	61	97.8	44
	F	1.8	2	1.6	1	2.2	1
A person should immediately go to a primary health care clinic after a NSI.	T	91.5	98	88.7	55	95.6	43
	F	8.4	9	1.3	7	4.4	2
Blood-borne infections can be transmitted by a NSI.	T	100.0	107	100.0	62	100.0	45
	F	0.0	0	0.0	0	0	0
Hepatitis B and C and HIV are the blood-borne pathogens to which healthcare providers are most commonly exposed when experiencing NSIs.	T	100.0	107	100.0	62	100.4	45
	F	0.0	0	0.0	0	0	0
Blood-borne infections can be transmitted through a sterile needle one that has not come in contact with another person during a NSI.	T	13.0	14	9.7	6	17.8	8
	F	8.6	93	90.3	56	82.2	37
Post exposure prophylaxis is a preventive medical treatment initiated after exposure to a pathogen to prevent infection.	T	93.4	100	95.2	59	91.2	42
	F	6.5	7	4.8	3	8.8	4
Antiretroviral drugs are mandatory immediately after a NSI has occurred.	T	66.3	71	62.9	39	71.2	32
	F	33.6	36	37.1	23	28.8	13
It is necessary to report a NSI if the laboratory result was negative.	T	85.0	91	77.4	48	95.6	43
	F	14.9	16	22.6	14	4.4	2

T, True; F, False; NSI, needle stick injury; HIV, human immunodeficiency virus.

### Chiropractic student respondents’ attitudes and perceptions of needle stick injuries and related protocols

The responses indicate that chiropractic students generally take NSIs seriously, with the majority disagreeing that NSIs are not something to worry about (75.6%; *n* = 81). Reporting NSIs is widely supported, with strong agreement, particularly among postgraduates (73.3%, *n* = 33). Most students, especially undergraduates (80.6%, *n* = 50), recognised the importance of vaccination against HBV. There was overwhelming agreement that NSIs should be reported regardless of fear of blame (75.7%, *n* = 81) and that PEP is necessary (81.2%, *n* = 87). While students agreed that NSI protocols should be practised (72.8%, *n* = 78) and that they should know how to report an NSI (78.5%, *n* = 84), there was less consensus on the importance of reporting NSIs from unused or sterilised needles (18.7; *n* = 20). Further details are presented in Table 3.

**TABLE 3:** Chiropractic student respondents’ attitudes and perceptions of needle stick injury and its related protocols.

Item	Level of agreement	Total sample		Undergraduate		Postgraduate	
		%	*n*	%	*n*	%	*n*
Needle stick injuries are not something to worry about.	Strongly disagree	2.8	3	0.2	1	4.0	2
	Disagree	4.6	5	6.4	4	2.2	1
	Neutral	1.7	18	20.9	13	11.1	5
	Agree	48.5	52	43.5	27	55.5	25
	Strongly agree	27.1	29	27.4	17	26.6	12
A NSI should be reported.	Strongly disagree	0.0	0	0.0	0	0.0	0
	Disagree	0.0	0	0.0	0	0.0	0
	Neutral	5.0	5	4.8	3	4.4	2
	Agree	30.8	33	37.0	23	22.2	10
	Strongly agree	64.4	69	58.0	36	73.3	33
It is important to be vaccinated against Hepatitis B.	Strongly disagree	0.9	1	0.0	0	2.0	1
	Disagree	0.0	0	0.0	0	0.0	0
	Neutral	7.0	7	4.8	3	8.8	4
	Agree	21.4	23	14.5	9	31.1	14
	Strongly agree	71.0	76	80.6	50	57.7	26
A NSI should be reported regardless of fear of blame or reprimanding.	Strongly disagree	0.0	0	0.0	0	0.0	0
	Disagree	0.0	0	0.0	0	0.0	0
	Neutral	6.0	6	6.4	4	4.4	2
	Agree	18.6	20	19.3	12	17.7	8
	Strongly agree	75.7	81	74.1	46	77.7	35
A NSI is a medical emergency.	Strongly disagree	1.8	2	0.0	0	4.0	2
	Disagree	13.0	14	12.9	8	13.3	6
	Neutral	28.0	30	30.6	19	24.4	11
	Agree	29.9	32	29.0	18	31.1	14
	Strongly agree	27.1	29	27.4	17	26.6	12
Post exposure prophylaxis, a preventative measure for infection, is necessary after exposure to a NSI.	Strongly disagree	0.9	1	0.0	0	2.0	1
	Disagree	0.9	1	1.6	1	0.0	0
	Neutral	17.0	18	16.1	10	17.7	8
	Agree	39.2	42	37.0	23	42.2	19
	Strongly agree	42.0	45	45.1	28	37.7	17
The needle stick protocol should be practised.	Strongly disagree	0.0	0	0.0	0	0.0	0
	Disagree	0.0	0	0.0	0	0.0	0
	Neutral	7.0	7	6.4	4	6.6	3
	Agree	20.5	22	22.5	14	17.7	8
	Strongly agree	72.8	78	70.9	44	75.5	34
Chiropractic students should know how to report a NSI.	Strongly disagree	0.0	0	0.0	0	0.0	0
	Disagree	1.8	2	3.2	2	0.0	0
	Neutral	0.0	0	0.0	0	0.0	0
	Agree	19.6	21	20.9	13	17.7	8
	Strongly agree	78.5	84	75.8	47	82.2	37
An NSI from an unused or sterilised needle should still be reported.	Strongly disagree	5.6	6	3.0	2	9.0	4
	Disagree	26.1	28	19.3	12	35.5	16
	Neutral	21.0	23	22.5	14	20.0	9
	Agree	28.0	30	30.6	19	24.4	11
	Strongly agree	18.6	20	24.1	15	11.1	5

NSI, needle stick injury.

As seen in Table 4, certain trends are observed between the undergraduate and postgraduate students. The postgraduate students showed a higher overall knowledge score (92.8%) compared to undergraduates (85.4%), indicating a better understanding of NSI protocols among the more experienced postgraduate cohort (*p* = 0.000). Despite this, variability was still noted within both groups, with some students scoring as low as 64.0%. Regarding attitudes and perceptions, both the undergraduate and postgraduate students demonstrated a general agreement with key statements, such as the importance of reporting NSIs (mean = 4.60) and vaccination against HBV (mean = 4.62). However, undergraduates showed a slightly higher mean score on the importance of HBV vaccination (mean = 4.76 for undergraduates compared to mean = 4.42 for postgraduates) placing greater importance on the need for HBV vaccination (*p* = 0.021). Other significant differences were observed in certain responses, such as the perception of whether NSIs from sterilised needles should be reported (*p* = 0.010) where postgraduates scored significantly lower on this item (mean = 2.93) than undergraduates (mean = 3.53).

**TABLE 4:** Mean, standard deviation and minimum and maximum range values for the chiropractic student respondents’ attitudes and perceptions of needle stick injury and its related protocols.

Item	Descriptives	Total sample	Undergraduate	Postgraduate
**Section B –** Overall percentage of true or false knowledge questions that were correct	*n*	107	62	45
	Mean (s.d.)	88.5 (± 9.455)	85.4 (± 8.958)	92.8 (± 8.479)
	Range	64–100	64–100	71–100
**Section C –** Overall attitude and perceptions towards NSI and related protocols	*n*	107	62	45
	Mean (s.d.)	4.26 (± 0.421)	4.30 (± 0.394)	4.22 (± 0.458)
	Range	2.89–5.00	3.44–5.00	2.89–5.00
**Section C –** Individual items				
Needle stick injuries are not something to worry about.	*n*	107	62	45
	Mean (s.d.)	3.93 (± 0.939)	3.89 (± 0.943)	3.98 (± 0.941)
	Range	1.00–5.00	1.00–5.00	1.00–5.00
A NSI should be reported.	*n*	107	62	45
	Mean (s.d.)	4.60 (± 0.580)	4.53 (± 0.593)	4.69 (± 0.557)
	Range	3.00–5.00	3.00–5.00	3.00–5.00
It is important to be vaccinated against Hepatitis B.	*n*	107	62	45
	Mean (s.d.)	4.62 (± 0.696)	4.76 (± 0.534)	4.42 (± 0.839)
	Range	1.00–5.00	3.00–5.00	1.00–5.00
A NSI should be reported regardless of fear of blame or reprimanding.	*n*	107	62	45
	Mean (s.d.)	4.70 (± 0.570)	4.68 (± 0.594)	4.73 (± 0.539)
	Range	3.00–5.00	3.00–5.00	3.00–5.00
A NSI is a medical emergency.	*n*	107	62	45
	Mean (s.d.)	3.67 (± 1.071)	3.71 (± 1.014)	3.62 (± 1.154)
	Range	1.00–5.00	2.00–5.00	1.00–5.00
Post exposure prophylaxis, a preventative measure for infection, is necessary after exposure to a NSI.	*n*	107	62	45
	Mean (s.d.)	4.21 (± 0.821)	4.26 (± 0.788)	4.13 (± 0.869)
	Range	1.00–5.00	2.00–5.00	1.00–5.00
The needle stick protocol should be practiced.	*n*	107	62	45
	Mean (s.d.)	4.66 (± 0.598)	4.65 (± 0.603)	4.69 (± 0.596)
	Range	3.00–5.00	3.00–5.00	3.00–5.00
Chiropractic students should know how to report a NSI.	*n*	107	62	45
	Mean (s.d.)	4.75 (± 0.551)	4.69 (± 0.642)	4.82 (± 0.387)
	Range	2.00–5.00	2.00–5.00	4.00–5.00
An NSI from an unused or sterilised needle should still be reported.	*n*	107	62	45
	Mean (s.d.)	3.28 (± 1.204)	3.53 (± 1.155)	2.93 (± 1.195)
	Range	1.00–5.00	1.00–5.00	1.00–5.00

NSI, needle stick injury; s.d., standard deviation.

## Discussion

The study demonstrated that both undergraduate and postgraduate chiropractic student respondents at the University of Johannesburg possess a good understanding of NSIs and related protocols. Most students accurately identified essential aspects of NSIs, including their definition, preventability and the importance of immediate reporting and proper needle disposal. However, postgraduates (mean percent of 92.9%) displayed a statistically significant higher percentage of correct responses in NSI-related practices compared to their undergraduate counterparts (mean percent of 85.5%). This aligns with existing literature that highlights undergraduate students, particularly those in their formative years of study, are at greater risk for NSIs, likely because of inexperience from minimal exposure to NSI training (Datar et al. 2022; Hambridge 2022; Musekene et al. 2020). The variability of these scores suggests that the current chiropractic education curricula and training on NSI protocols at this institution may not be consistent throughout the years of the BHSc and MHSc qualifications, presenting a critical need for improvement to address knowledge gaps that persist across all educational levels.

The study also revealed a marked difference in the promotion of active bleeding after a NSI between the two groups. While two-thirds (67.7%) of undergraduate students disagreed with the practice of promoting bleeding, a significant majority of postgraduates (86.7%) endorsed it. This divergence suggests a gap in understanding or acceptance of post-exposure protocols, which are crucial for effective NSI management. Furthermore, 69.4% of undergraduates correctly identified that washing the affected area with soap and water is recommended, compared to 95.6% of postgraduates who answered correctly. This finding is consistent with the practices reported by in other studies (Goel et al. 2017; Jahangiri et al. 2016), which noted that washing the site with soap and water is the most common and generally accepted as most correct post-exposure action among healthcare professionals. However, the National Health Service recommends a more comprehensive approach (NHS 2022), encouraging the wound to bleed while preferably washing it thoroughly with soap and water and finally covering it with a waterproof dressing. This protocol emphasises the importance of promoting bleeding as a critical first step, a practice more commonly adhered to by postgraduate students, suggesting that their more advanced training may contribute to a better understanding of effective NSI management (NHS 2022). These differing practices highlight a potential gap in the standardisation of the NSI training offered. To avoid suboptimal post-exposure activity, NSI training within chiropractic curriculum should standardise and reinforce evidence-based NSI management protocols to improve overall safety in clinical settings.

Attitudinal differences were also observed between the groups, with postgraduates showing greater support for the immediate reporting of NSIs and stronger adherence to established protocols. While knowledge of NSI-related risks, such as HBV and HCV and HIV, was unanimous among all respondents, undergraduates placed greater emphasis on the importance of HBV vaccination. Previous studies have shown that medical students often perceive themselves as being at higher risk for contracting and spreading HBV but may lack sufficient knowledge about PEP (Al-Hazmi 2015). Similarly, a 2018 study found that, despite a good general understanding of HIV and HBV transmission, medical students often lacked specific knowledge about PEP guidelines, duration and use (Patel et al. 2018). This finding suggests that postgraduate chiropractic students place less emphasis on HBV vaccination, possibly as they perceive vaccination as routine procedure rather than a critical preventive measure, which allows an opportunity to communicate the importance of vaccination throughout training stages.

Another significant finding was the variation in attitude towards the reporting of an NSI involving an unused sterile needle and the misconception among undergraduates that sterile needles could transmit infections, indicating a lack of consensus among students about the necessity of reporting NSIs from unused sterile needles. While NSIs involving unused sterile needles are not considered to pose any infectious risk (Suksatan et al. 2022), the reporting procedure of any and all NSIs, regardless of needle condition, should be followed to ensure adequate risk assessment (Alsabaani et al. 2022). These findings suggest that postgraduate students possibly underestimate the importance of reporting all NSIs. Chiropractic curriculum and NSI protocol training should extend into the importance of reporting and monitoring of NSIs regardless of needle condition.

Overall, while both undergraduate and postgraduate students are generally well informed about NSIs, the study highlighted specific areas where NSI education and training in chiropractic could benefit from earlier and more standardised integration into the curriculum. The significant knowledge and attitude differences between the two groups suggest that tailored educational interventions are necessary to ensure all students are equally prepared throughout the various years of study. Evidence from Taiwan, the United States of America and China demonstrates the effectiveness of year-specific educational programmes in significantly reducing NSI incidence among healthcare students. Implementing similar interventions in chiropractic education could improve student safety and the quality of care they provide (Reid et al. 2014; Yang et al. 2007; Yao et al. 2013). Based on the overall findings of this study, there is a need for future research that should consider the long-term knowledge retention of NSI protocols among students through longitudinal studies, the effectiveness of targeted NSI training, the possible psychological and sociocultural factors that may influence students’ NSI reporting and investigate students’ implementation and compliance practices of NSI protocols.

### Study limitations

The study’s limitations include a specific sample that limits generalisability. The reliance on self-reported data is prone to bias. A cross-sectional design prevents assessing changes that may occur over time.

## Conclusion

This study highlights the favourable knowledge of NSIs and related protocols among both undergraduate and postgraduate chiropractic students at University of Johannesburg. However, significant differences in attitudes and perceptions between the two cohorts emphasise the need for targeted and standardised NSI educational interventions. Postgraduates demonstrated a higher level of awareness and adherence to recommended practices, particularly regarding the management of NSIs and the importance of immediate reporting and post-exposure actions. In contrast, undergraduates, especially those in the earlier years of study, showed gaps in knowledge and misconceptions that could increase their risk of NSIs. Varied views among the postgraduate and undergraduate chiropractic students were also highlighted regarding the importance on vaccination and reporting of unused sterile NSIs. Addressing these gaps through tailored and standardised educational programmes based on the year of study has the potential to significantly reduce NSI incidence and improve overall safety among chiropractic students at the University of Johannesburg. Implementing such interventions could lead to better preparedness and safer practices, ultimately enhancing the quality of care provided by future healthcare professionals.
